# Synthesis and Electrocatalytic Performance Study of Sulfur Quantum Dots Modified MoS_2_

**DOI:** 10.3390/molecules29112551

**Published:** 2024-05-29

**Authors:** Guiyu Wei, Tao Tang, Ruizheng Xu, Zhemin Xie, Sijie Diao, Jianfeng Wen, Li Jiang, Guanghui Hu, Ming Li

**Affiliations:** 1 Key Laboratory of Low-Dimensional Structural Physics and Application, Education Department of Guangxi Zhuang Autonomous Region, College of Physics and Electronic Information Engineering, Guilin University of Technology, Guilin 541004, China; wgy1020211714@163.com (G.W.); 18816704460@163.com (R.X.); xiezhemin0420@163.com (Z.X.); jiniaohuaguyuan717@163.com (S.D.); 2005026@glut.edu.cn (J.W.); jiangli1515@glut.edu.cn (L.J.); 2 School of Electronic Information and Automation, Guilin University of Aerospace Technology, Guilin 541004, China; tangtao@glut.edu.cn

**Keywords:** molybdenum disulfide, sulfur quantum dots, hydrogen evolution reaction, electrocatalysis

## Abstract

The electrolysis of water for hydrogen production is currently receiving significant attention due to its advantageous features such as non-toxicity, safety, and environmental friendliness. This is especially crucial considering the urgent need for clean energy. However, the current method of electrolyzing water to produce hydrogen largely relies on expensive metal catalysts, significantly increasing the costs associated with its development. Molybdenum disulfide (MoS_2_) is considered the most promising alternative to platinum for electrocatalyzing the hydrogen evolution reaction (HER) due to its outstanding catalytic efficiency and robust stability. However, the practical application of this material is hindered by its low conductivity and limited exposure of active sites. MoS_2_/SQDs composite materials were synthesized using a hydrothermal technique to deposit SQDs onto MoS_2_. These composite materials were subsequently employed as catalysts for the HER. Research findings indicate that incorporating SQDs can enhance electron transfer rates and increase the active surface area of MoS_2_, which is crucial for achieving outstanding catalytic performance in the HER. The MoS_2_/SQDs electrocatalyst exhibits outstanding performance in the HER when tested in a 0.5 M H_2_SO_4_ solution. It achieves a remarkably low overpotential of 204 mV and a Tafel slope of 65.82 mV dec^−1^ at a current density of 10 mA cm^−2^. Moreover, during continuous operation for 24 h, the initial current density experiences only a 17% reduction, indicating high stability. This study aims to develop an efficient and cost-effective electrocatalyst for water electrolysis. Additionally, it proposes a novel design strategy that uses SQDs as co-catalysts to enhance charge transfer in nanocomposites.

## 1. Introduction

With the rapid advancement of human civilization, there is an increasing dependence on various forms of energy. Fossil fuels remain a predominant and indispensable energy source in the contemporary world. However, due to the limited availability of fossil fuels and the steadily growing global demand, their scarcity or complete depletion is inevitable [1]. Furthermore, the extensive use of fossil fuels has led to significant degradation of the ecological environment [2,3]. Therefore, the advancement of clean and sustainable energy sources represents a significant challenge for the international community currently [4,5]. Hydrogen has garnered considerable attention as an energy carrier due to its exceptionally high energy density, purity, and renewable characteristics [6]. However, current methods for hydrogen production still heavily rely on fossil fuels, thus inadequately addressing this issue at its root. Alternatively, renewable energy sources can be used to produce high-purity hydrogen sustainably via water electrolysis [7]. However, the efficiency of water electrolysis is significantly reduced due to slow hydrogen evolution reaction kinetics, resulting in a significant increase in costs [8,9]. Noble metal materials, such as platinum-based catalysts, exhibit outstanding electrocatalytic performance for the HER. However, their high cost and limited availability hinder the widespread adoption of these materials. To reduce dependence on platinum-based catalysts, it is crucial to develop novel, cost-effective, and efficient catalysts for the HER that are free from precious metals.

Researchers have thus far concentrated on investigating various transition metal-based materials as potential catalysts for the HER that exhibit exceptional performance [10]. Transition metal-based compounds, particularly those in Group VI B like molybdenum and tungsten, serve as substantial alternatives to precious metals for catalyzing the HER process. Molybdenum-based materials have exhibited excellent performance in the HER, attracting considerable research attention in recent years. Molybdenum-containing compounds exhibit exceptional performance, leading to their extensive application across diverse disciplines, including molecular probes [11], electrochemical capacitors [12], catalysts [13,14], and batteries [15,16]. The investigation into electrochemical HER materials utilizing molybdenum has been ongoing for several decades. Recently, there has been a surge in research exploring various types of molybdenum-based compounds, including alloys, sulfides, selenides, carbides, phosphides, borides, nitrides, and oxides, as highly efficient catalysts for HER. These compounds exhibit substantial electrocatalytic activity and stability across a wide pH range, encompassing strong acids, strong alkalis, and neutral solutions [17]. Transition metal dichalcogenides have become widely recognized as effective electrocatalysts for catalyzing the HER in acidic environments. This is mostly due to their exceptional catalytic efficiency and stability, making them a promising alternative to precious metal catalysts [18,19]. MoS_2_, one of the transition metal dichalcogenides (TMDs), is a stable and non-toxic material with a low cost. It shows great promise in the field of electrocatalysis for the HER [20,21]. However, MoS_2_, extensively researched as a potential alternative to platinum-based HER electrocatalysts, encounters challenges due to its low electrical conductivity and limited accessibility of active sites, which hinder its practical application [22]. Several approaches have been proposed to enhance the reactivity of MoS_2_, such as enhancing the exposure of active sites, combining it with highly conductive materials, and introducing metal atoms to enhance the intrinsic reactivity of its edges [23,24,25]. For example, research has documented the utilization of Cr-doped FeNi-P nanoparticles and N-doped CoP nanoarrays, both exhibiting significant improvements in their HER capabilities upon the incorporation of chromium (Cr) or nitrogen (N) into the transition metals [26,27]. However, relying solely on elemental doping is insufficient to surpass the performance of Pt and Pt-based materials. Additionally, the presence of a substrate is crucial to offer structural support for transition metal materials [28]. Chen et al. found that incorporating carbon quantum dots (CQDs) onto transition metal phosphide surfaces enhances their performance in the HER by increasing the surface area and the number of active sites [27]. Typically, small 0D nanometer-sized particles (less than 10 nanometers) offer inherent advantages that facilitate their integration into various material structures. The utilization of nanodots, such as CQDs and graphene quantum dots, can significantly enhance electron mobility. Sulphur quantum dots (SQDs) are attracting attention as a promising area of study in electrochemistry, owing to their distinctive characteristics and versatile applications. Nanoscale semiconductor materials typically range in dimensions from 1 to 10 nanometers and exhibit intriguing electrochemical properties due to their adjustable bandgaps, high surface area-to-volume ratio, and exceptional charge transport capabilities. SQDs, or semiconductor quantum dots, demonstrate significant potential across various areas such as energy storage, catalysis, and sensing within the field of electrochemistry. SQDs exhibit high efficiency in conducting redox processes, making them highly suitable for application in batteries, supercapacitors, and electrocatalysis. To date, there are no documented instances of molybdenum-based catalysts being utilized in conjunction with SQDs for HER.

The objective of this study is to attach sulfur quantum dots onto molybdenum disulfide to enhance its electrocatalytic hydrogen evolution performance. This will be achieved by creating a synergistic catalytic system. The incorporation of sulfur quantum dots has a dual effect: improving the conductivity of molybdenum disulfide and facilitating electron transport. Additionally, it regulates the active sites on the surface of molybdenum disulfide, leading to an enhancement in its electrocatalytic hydrogen evolution capability. Sulfur quantum dots and molybdenum disulfide were synthesized via a hydrothermal process, and composite materials of sulfur quantum dots and molybdenum disulfide were effectively analyzed using various techniques. Subsequently, the electrochemical efficiency of hydrogen production was individually assessed for sulfur quantum dots/molybdenum disulfide, pure molybdenum disulfide, and sulfur quantum dots in an acidic electrolyte. The experimental results demonstrate that the HER performance of the sulfur quantum dots/molybdenum disulfide composite surpasses that of both pure MoS_2_ and sulfur quantum dots alone, exhibiting a lower onset potential and Tafel slope. The composite material exhibits exceptional durability during stability tests. These results suggest that combining sulfur quantum dots with molybdenum disulfide is a successful approach to improving the electrocatalytic performance of the hydrogen evolution reaction.

## 2. Results and Discussion

### 2.1. Structure Characterization

**Figure 1:**
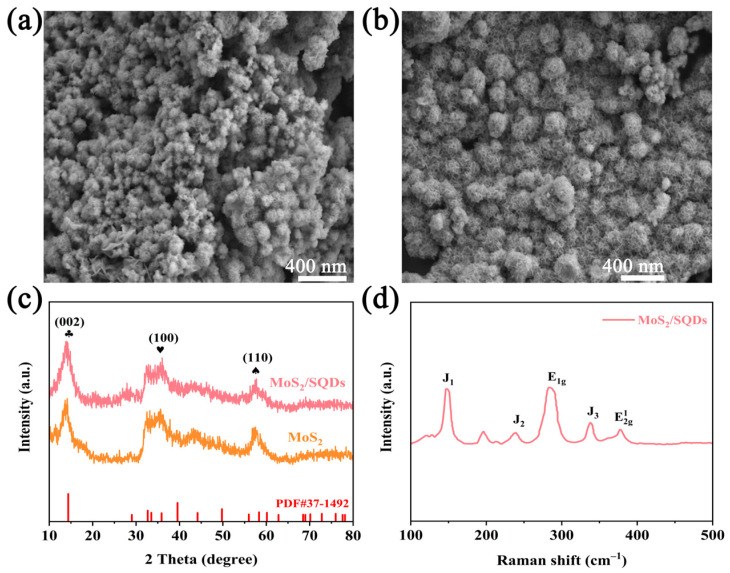
(**a**,**b**) SEM of MoS_2_ and MoS_2_/SQDs; (**c**) XRD of MoS_2_ and MoS_2_/SQDs; (**d**) Raman spectra of MoS_2_/SQDs.

To gain a more comprehensive understanding and conduct a detailed analysis of the structure of MoS_2_, both before and after the incorporation of SQDs, scanning electron microscopy was employed to investigate the morphology of MoS_2_. The SEM images of pure MoS_2_ and MoS_2_/SQDs are depicted in Figure 1a,b, respectively. These images clearly illustrate the regular and closely arranged structure of MoS_2_, resembling a rose-like formation. The addition of SQDs has enhanced the aggregation of molybdenum disulfide compared to pure MoS_2_, resulting in a loosely structured composition comprising several tiny nanosheets. MoS_2_/SQDs exhibit a greater number of exposed edges compared to pure MoS_2_, potentially leading to an increased number of active sites available for the HER and consequently enhancing the electrocatalytic activity of MoS_2_. The XRD graph (Figure 1c) reveals that the diffraction peaks of MoS_2_ closely match those of standard MoS_2_ in the PDF card (PDF#37-1492) [28]. Specifically, the (002), (100), and (110) crystal plane peaks are observed at angles of 14.3°, 32.6°, and 58.6°, respectively. The displacement of the diffraction peaks in MoS_2_/SQDs, compared to pure MoS_2_, is nearly insignificant. Figure 1d displays the $E_{2g}^{1}$ vibration of MoS_2_/SQDs at 377.46 cm^−1^, corresponding to in-plane Mo-S vibration, while the E_1g_ peak at 284.30 cm^−1^ is associated with octahedrally coordinated Mo atoms. Additionally, peaks J_1_, J_2_, and J_3_ at 147.59, 238.73, and 337.97 cm^−1^, respectively, correspond to the superlattice distortion of the MoS_2_ basal plane [29].

**Figure 2:**
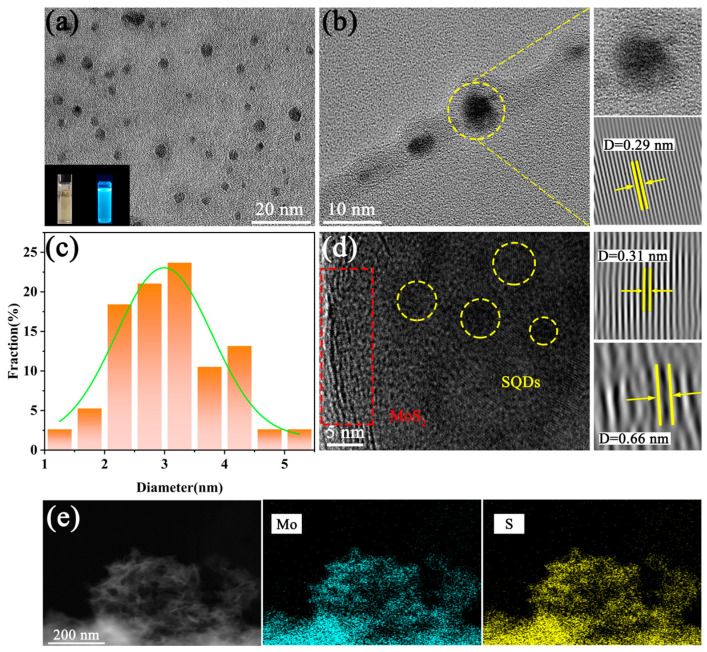
(**a**,**b**) TEM images of SQDs; (**c**) size distribution of SQDs; (**d**,**e**) TEM images and elemental mapping of MoS_2_/SQDs.

The TEM technique was employed for further characterization. Figure 2a,b present the microstructure of SQDs. Figure 2a illustrates the uniformly distributed and highly spherical crystalline structures of all SQDs. In Figure 2b, a lattice spacing of 0.29 nm in the SQDs is clearly visible, corresponding to the (243) crystal plane. Figure 2c provides the size distribution of SQDs, indicating a size range of 1 nm to 5 nm, directly correlating with the size of the SQDs. TEM images confirm the presence of both MoS_2_ and SQDs in the sample (Figure 2d). The lattice spacings of 0.31 nm and 0.66 nm observed in the TEM images correspond to the (002) crystal plane of MoS_2_ and the (243) plane of SQDs, respectively. Additionally, the EDS spectra demonstrate the homogeneous dispersion of Mo and S elements (Figure 2e).

**Figure 3:**
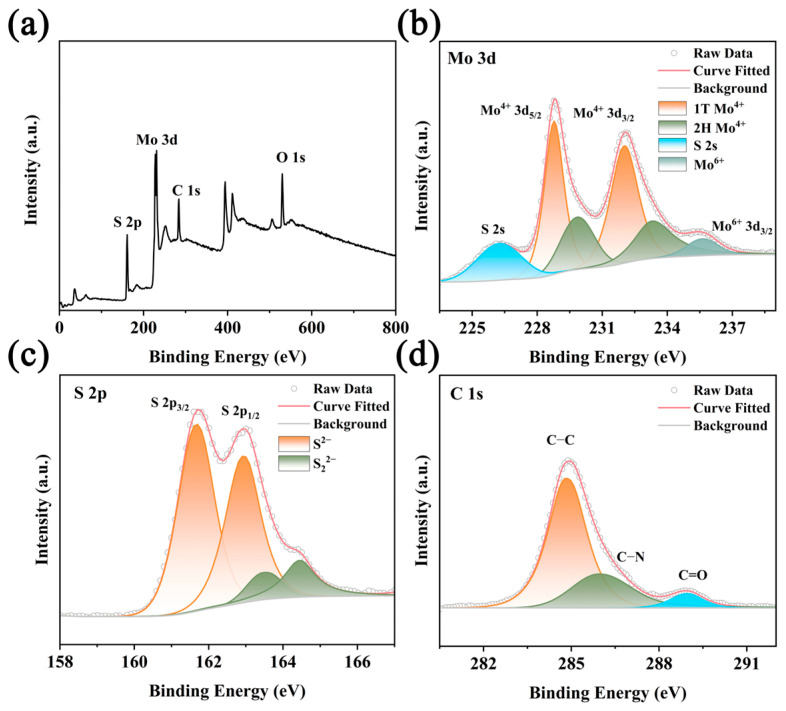
XPS spectra of MoS_2_/SQDs. (**a**) Full spectrum; (**b**) Mo 3d; (**c**) S 2p; (**d**) C 1s.

The surface composition and valence states of the synthesized MoS_2_/SQDs samples were determined using X-ray photoelectron spectroscopy (XPS). The XPS scans revealed the presence of sulfur (S), molybdenum (Mo), oxygen (O), and carbon (C) components in the samples (Figure 3a). The Mo 3d spectrum (Figure 3b) shows six prominent peaks: the peaks at 228.75 and 232.02 eV are identified as the Mo 3d_3/2_ of Mo^4+^, while the peaks at 229.86 and 233.33 eV correspond to the Mo 3d_5/2_ of Mo^4+^. The peak at a binding energy of 226.24 eV is attributed to S 2s, and the peak at a binding energy of approximately 235.5 eV is the Mo 3d_3/2_ peak of Mo^6+^, consistent with previous research findings [30]. From Figure 3c, it can be observed that there are two strong peaks located at 161.7 eV and 162.9 eV in the high-resolution spectra of S 2p_1/2_ and S 2p_3/2_, respectively, and the sulfur species exist in the form of S^2−^ ions [31]. Based on the fitting results, the peak observed at 161.5 eV likely indicates the presence of sulfur ions in a condition of low coordination on the surface, while the peak at 163.1 eV is indicative of the molybdenum–sulfur bond in MoS_2_. The peaks observed at 284.8, 286.3, and 288.9 eV in the C 1s spectra (Figure 3d) correspond to the presence of C-C, C-N, and C=O bonds, respectively.

### 2.2. Electrochemical Performance Analysis

**Figure 4:**
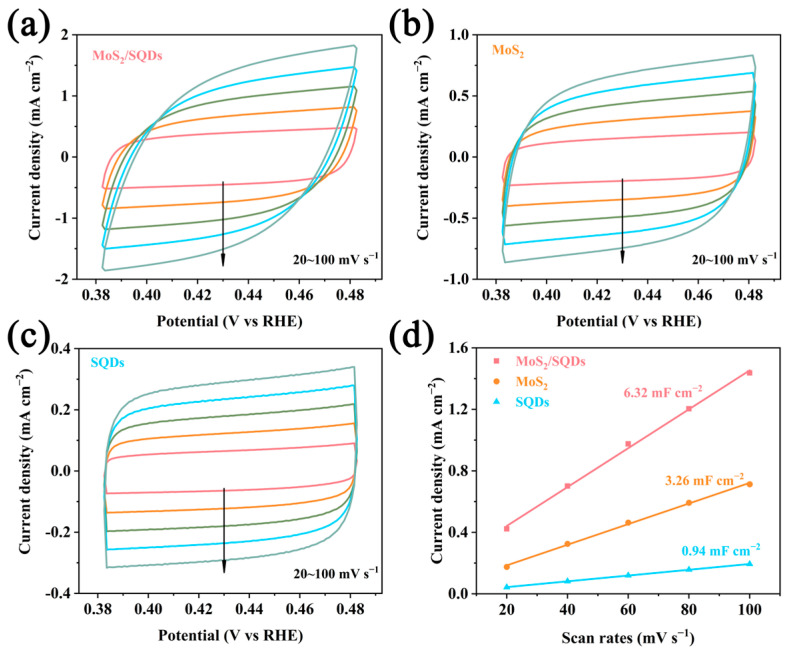
The CV curves of MoS_2_/SQDs, MoS_2_, and SQDs are represented by (**a**), (**b**), and (**c**), respectively; (**d**) illustrates the capacitance double-layer diagram of MoS_2_/SQDs.

The number of active sites on the catalyst was assessed using double-layer capacitance analysis, while cyclic voltammetry was employed to analyze the electrochemical performance of the synthesized materials. Figure 4a–c depict the CV curves and the linear correlation between current density and scan rate at various scan rates. In comparison to other samples, MoS_2_/SQDs demonstrate a higher current density in the CV curves, suggesting a higher specific capacitance (Figure 4d). The obtained results indicate that the capacitance values of self-made MoS_2_ and MoS_2_/SQDs are 3.26 mF cm^−2^ and 6.32 mF cm^−2^, respectively. The charge carrier density of MoS_2_/SQDs is approximately double that of pure MoS_2_, indicating a greater number of active sites. This phenomenon can be attributed to the dispersion of previously clustered MoS_2_ nanosheets by SQDs, leading to a significant increase in the active surface area of MoS_2_ and consequently creating additional sites for chemical reactions, thereby enhancing overall reaction rates.

**Figure 5:**
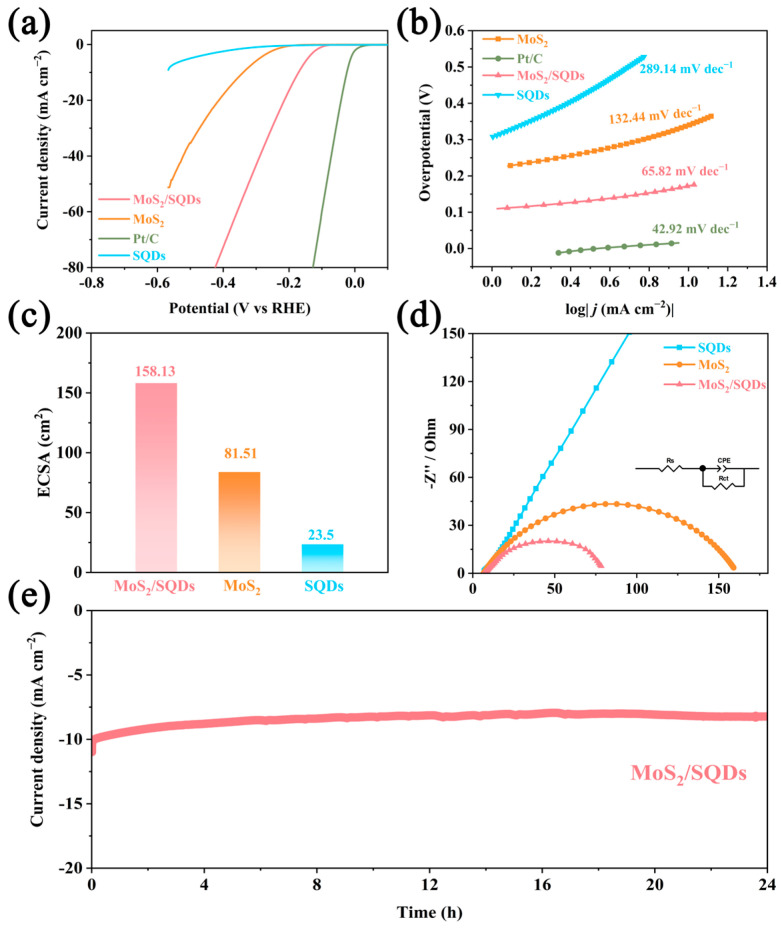
The electrochemical characteristics of each sample were evaluated using the following methods: (**a**) linear scanning voltammetry; (**b**) Tafel curve analysis; (**c**) estimation of the electrochemical active area; (**d**) measurement of the electrochemical impedance spectra; (**e**) assessment of the stability of the MoS_2_/SQDs samples over a 24 h period.

The electrochemical performance of the prepared samples for HER was evaluated in an acidic environment using a three-electrode electrochemical workstation, with a scan rate of 1 mV s^−1^. Figure 5a illustrates the linear sweep voltammetry profiles of commercially available 20% Pt/C, MoS_2_, SQDs, and MoS_2_/SQDs electrodes. While the Pt/C catalyst exhibits anticipated HER activity with an overpotential almost at zero, SQDs, however, perform poorly within the test potential range, suggesting minimal catalytic action for HER. Remarkably, MoS_2_/SQDs demonstrate stronger catalytic activity than pure molybdenum disulfide. The overpotential of MoS_2_ is 340 mV at a current density of 10 mA cm^−2^, much higher than that of MoS_2_/SQDs (204 mV). This suggests that MoS_2_ is the primary active component for HER, as indicated by the lower catalytic activity of SQDs. The combination of MoS_2_ and SQDs significantly enhances the catalytic performance of MoS_2_/SQDs in the HER process.

To comprehend the HER mechanism, we determined Tafel slopes by fitting the linear portion of the Tafel plots. Figure 5b depicts that the Tafel slope of the MoS_2_/SQDs catalyst is 65.82 mV dec^−1^, lower than that of MoS_2_ (132.44 mV dec^−1^) and SQDs (289.14 mV dec^−1^), indicating higher HER rates and faster kinetics for the MoS_2_/SQDs catalyst. A Tafel slope value of 65.82 mV dec^−1^ suggests the occurrence of the Volmer–Heyrovsky mechanism on MoS_2_/SQDs during the HER process, where the Heyrovsky step dictates the reaction rate. Combining SQDs with MoS_2_ improves the separation of water molecules in the Volmer step, leading to increased HER kinetics and faster rates of charge transfer.

The ECSA of catalysts is determined by estimating the catalyst’s electrochemical C_dl_, measured using CV in the non-Faradaic zone with gradually increasing scan rates. Among the MoS_2_/SQDs, MoS_2_, and SQDs samples, the MoS_2_/SQDs sample exhibits the highest C_dl_ value at 6.32 mF cm^−2^. The ECSA of the MoS_2_/SQDs catalyst is the greatest among the prepared catalysts, calculated to be 158.13 cm^2^ (Figure 5c). In comparison, the ECSA values for pure MoS_2_ and SQDs are calculated to be 81.51 cm^2^ and 23.5 cm^2^, respectively.

Figure 5d illustrates the electrochemical impedance spectra of the materials. The Nyquist plot commonly uses the diameter of the semicircle to ascertain the charge transfer resistance. A reduced diameter corresponds to decreased charge transfer resistance and an accelerated charge transfer rate. The inclusion of SQDs decreases the electrical resistance of MoS_2_, as indicated by notably lower semicircle diameters observed in MoS_2_/SQDs compared to MoS_2_ and SQDs alone. This reduction in resistance is beneficial for lowering the resistance of the interface reaction and enhancing performance, facilitating quicker electron transfer between the catalyst and electrolyte, thereby increasing charge transport during the HER process.

**Figure 6:**
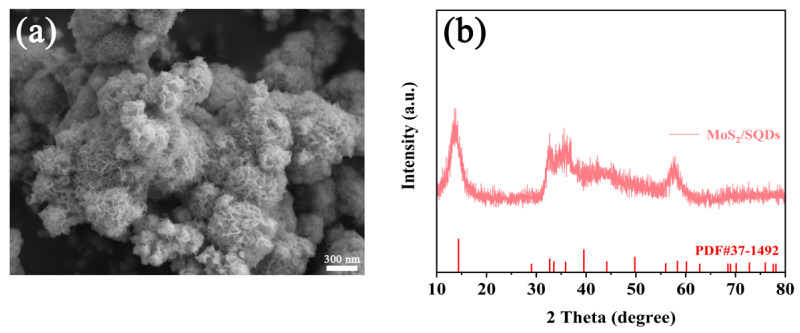
SEM (**a**) and XRD (**b**) of MoS_2_/SQDs after 24 h electrochemical stability testing.

Stability is a significant criterion for catalysts, in addition to their catalytic activity. To further examine the stability of MoS_2_/SQDs in the electrocatalytic HER, current–time curves under overpotential conditions were measured. As depicted in Figure 5e, the initial current density of the MoS_2_/SQDs catalyst decreased by a mere 17% following a 24 h testing period. The exceptional resistance of MoS_2_/SQDs to electrochemical degradation in acidic environments was confirmed through a 24 h endurance test. The decrease in current density could potentially be explained by gas desorption on the electrode surface or sluggish mass transport of the electrolyte. As shown in Figure 6, after 24 h of stability testing, there were no significant changes in the phase and morphology of MoS_2_/SQDs, and they remained essentially consistent before and after stability testing. The high stability of MoS_2_/SQDs throughout prolonged electrochemical processes is validated by these results. Table 1 below demonstrates the comparison of the performance of MoS_2_/SQDs with other HER electrocatalysts.

## 3. Materials and Methods

### 3.1. Materials

Sodium molybdate dihydrate, thioacetamide (TAA), sulfuric acid, sublimed sulfur, polyethylene glycol (PEG), sodium hydroxide, anhydrous ethanol, and Nafion (5 wt%) were purchased from Shanghai Aladdin Bio-Chem Technology Co., Ltd. (Shanghai, China) and Pt/C (20 wt%) was purchased from Shanghai Hesen Electric Co., Ltd. (Shanghai, China). All reagents are ready for use without any further treatment.

### 3.2. Preparation of SQDs

SQDs were synthesized using a hydrothermal method [40]. First, 1.6 g of S, 3 g of PEG, 4 g of NaOH, and 100 mL of deionized water were stirred at 90 °C for 10 h. After cooling to room temperature, impurities were removed using a 0.22 µm pore size membrane. Dialysis was performed, and the SQD solution was collected after the dialysis was complete.

### 3.3. Preparation of MoS_2_

Next, 0.3 g of Na_2_MoO_4_·2H_2_O and 0.28 g of C_2_H_5_NS were sequentially added to 25 mL of deionized water. After magnetic stirring for 30 min, the mixture was transferred to a 50 mL high-pressure reaction vessel and subjected to hydrothermal reaction at 200 °C for 18 h. After natural cooling to room temperature, repeated centrifugation with a mixture of deionized water and anhydrous ethanol was performed to remove other impurity ions. Finally, the product was freeze-dried to collect the final product.

### 3.4. Preparation of MoS_2_/SQDs

**Figure 7:**
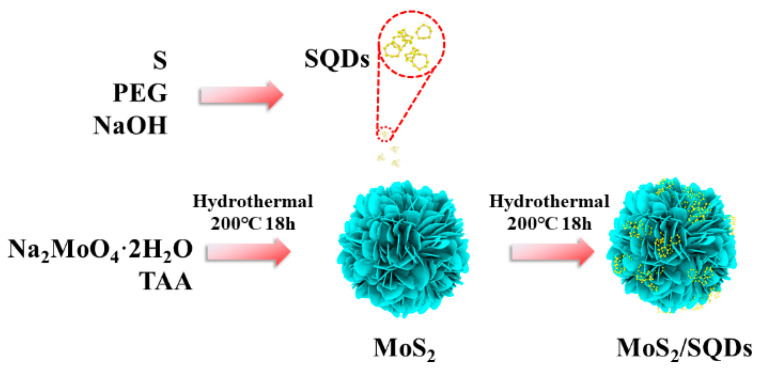
Preparation process scheme of MoS_2_/SQDs.

As shown in Figure 7, MoS_2_/SQDs were synthesized via the hydrothermal method, using Na_2_MoO_4_·2H_2_O as the molybdenum source and C_2_H_5_NS as the sulfur source. Specifically, 0.3 g of Na_2_MoO_4_·2H_2_O and 0.28 g of C_2_H_5_NS were sequentially added to 30 mL of deionized water. After magnetic stirring for 20 min, 20 mL of SQD solution was added, followed by transfer to a 100 mL high-pressure reaction vessel for hydrothermal reaction at 200 °C for 18 h. After natural cooling to room temperature, repeated centrifugation with a mixture of deionized water and anhydrous ethanol was performed to remove other impurity ions. Finally, the product was freeze-dried to collect the final product.

### 3.5. Characterization

X-ray diffraction (XRD; MiniFlex-600, Rigaku, Tokyo, Japan) with a CuKα source was used to analyze the crystalline condition of samples. X-ray photoelectron spectroscopy (XPS; K-Alpha, Thermo Scientific, Waltham, MA, USA) was used to analyze the elemental valence of materials. Transmission electron microscopy (TEM; JEM-2100, JEOL Ltd., Tokyo, Japan) was used to test the grain size of the samples. Scanning electron microscopy (SEM; KYKY-EM 6900, KYKY Technology Co., Ltd., Beijing, China) was utilized to observe the morphology of the samples. Fourier-transform infrared (FTIR; FTIR-2000, PerkinElmer, Waltham, MA, USA) spectrometry was employed to analyze the structure of the samples.

### 3.6. Electrochemical Measurements

Herein, electrochemical measurements were performed in a 0.5 M H_2_SO_4_ electrolyte using a standard three-electrode system (CHI 760E Instruments, Chenhua, Shanghai, China) with a Ag/AgCl reference electrode (saturated with KCl) as a reference, graphite rod as a counter electrode, and GCE (3 mm, diam.) as a working electrode. Data were corrected to reversible hydrogen electrode (RHE) without current and resistance (IR) modifications by employing the formula E(RHE) = E(Ag/AgCl) + 0.197 + 0.059pH. Cyclic voltammetry (CV) was executed over 200 cycles using a scan rate of 50 mV s^−1^ within the non-Faraday reaction voltage range, resulting in the acquisition of consistent CV curves. The scanning range of electrochemical double layer capacitance (C_dl_) was 0.15 to 0.25 V at various scanning rates (20, 40, 60, 80, and 100 mV s^−1^). Linear scanning voltammetry (LSV) was conducted with a scan rate of 1 mV s^−1^ over a voltage window from −0.6 to 0.1 V. Electrochemical impedance spectroscopy (EIS) measurements were conducted over a frequency interval from 0.01 Hz to 100 kHz with an amplitude of 5 mV. Electrochemical surface area (ECSA) was calculated based on CV curves using the formula ECSA = C_dl_/Cs, with Cs denoting the specific capacitance from 0.02 to 0.06 mF cm^−2^. A mean value of 0.04 mF cm^−2^ was adopted in this study.

## 4. Conclusions

An introduction was provided on a hydrothermal approach for preparing catalysts such as SQDs, MoS_2_, and MoS_2_/SQDs. The characterization results of the synthesized materials were analyzed in detail using SEM, Raman, XRD, XPS, and TEM, confirming the successful synthesis of MoS_2_/SQD composite samples. Subsequently, the electrocatalytic activity of the MoS_2_/SQD composite material for the HER was investigated and compared to SQDs and self-synthesized MoS_2_. The results revealed the notably superior catalytic activity of the MoS_2_/SQDs composite catalyst compared to the aforementioned catalysts. The addition of SQDs significantly enhances the electrocatalytic performance of the MoS_2_ catalyst, as evidenced by the characterization results for morphology, surface characteristics, and valence states of the elements. Molybdenum disulfide has a greater specific surface area and more edges, attributed to the presence of SQDs, leading to an increased number of catalytic active sites for the HER process. The research findings suggest that MoS_2_/SQDs have a reduced Tafel slope of 62.85 mV dec^−1^ and a decreased overpotential of 204 mV at 10 mA cm^−2^. Besides its outstanding catalytic activity, the MoS_2_/SQDs catalyst also exhibits remarkable stability, with only a 17% reduction in initial current density after undergoing 24 h of timed current stability testing at 10 mA cm^−2^. This presents a novel methodology for creating high-efficiency catalysts for the HER.

## Figures and Tables

**Table 1:** Comparison of MoS_2_/SQDs with other HER electrocatalysts.

Catalyst	*η*_10_ (mV)	Tafel (mV dec^−1^)	Electrolyte	Ref.
SiQDs-MoS_2_	200	60	0.5 M H_2_SO_4_	[32]
SnS_2_-MoS_2_	240	65	0.5 M H_2_SO_4_	[33]
Ni-MoS_2_	302	66.27	0.5 M H_2_SO_4_	[34]
MoS_2_/MoO_2_	235	64	0.5 M H_2_SO_4_	[35]
MoS_2_·ZnO	239	66	0.5 M H_2_SO_4_	[36]
Zn–MoS_2_	300	51	0.5 M H_2_SO_4_	[37]
MoS_2_/Co-MOF	262	51	0.5 M H_2_SO_4_	[38]
MoS_2_/3D-NPC	210	51	0.5 M H_2_SO_4_	[39]
MoS_2_/SQDs	204	65.82	0.5 M H_2_SO_4_	This work

## Data Availability

The data can be made available upon reasonable request.
